# The relationship between premorbid intelligence and symptoms of severe anorexia nervosa restricting type

**DOI:** 10.7150/ijms.53907

**Published:** 2021-02-04

**Authors:** Keizaburo Ogata, Ken Ichiro Koyama, Takamasa Fukumoto, Suguru Kawazu, Mihoko Kawamoto, Eriko Yamaguchi, Yuuki Fuku, Marie Amitani, Haruka Amitani, Ken Ichiro Sagiyama, Akio Inui, Akihiro Asakawa

**Affiliations:** 1 Division of Clinical Psychology, Kitasato University Hospital, Kanagawa, Japan.; 2 Department of Psychosomatic Internal Medicine, Kagoshima University Graduate School of Medical and Dental Sciences, Kagoshima, Japan.; 3 Faculty of Integrated Human Studies and Social Sciences, Fukuoka Prefectural University Graduate School of Human and Social Sciences, Fukuoka, Japan.; 4 Education Center for Doctors in Remote Islands and Rural Areas, Kagoshima University Graduate School of Medical and Dental Sciences, Kagoshima, Japan.; 5 Pharmacological Department of Herbal Medicine, Graduate School of Medical and Dental Sciences, Kagoshima University, Kagoshima, Japan.

**Keywords:** premorbid intelligence, anorexia nervosa

## Abstract

The purposes of this study were as follows: to compare premorbid IQ with present IQ in patients with more severe anorexia nervosa restricting type (AN-R) and to investigate the relationship between decreasing IQ and symptoms in patients with severe AN-R. Twenty-two participants were recruited (12 were AN-R patients; 10 were healthy controls). The average BMI in AN-R patients and healthy controls was 12.65 and 19.82, respectively. We assessed the outcomes using the Wechsler Adult Intelligence Scale-Third Edition (WAIS-III), the Japanese Adult Reading Test, The Eating Disorders Inventory-2 (EDI-2), Beck Depression Scale-2 (BDI-2) and State-Trait Anxiety Index. In two-way ANOVA, there were significant interactions for the FIQ and PIQ. Only in the AN-R group, a significant single main effect of time was evidenced for the FIQ and PIQ. In the AN-R group, a significantly high positive correlation was found between changes in the PIQ and the body dissatisfaction subscale of the EDI-2. These findings raise the possibility that in patients with severe AN-R, an excessive decrease in body weight induces decreased PIQ; as a result, they have worse dissatisfaction with their body shape.

## Introduction

Anorexia nervosa (AN) is a psychiatric disorder that pursues body weight loss 1,2. AN restricting type (AN-R) is characterized by severe emaciation with long-term food restriction, leading to behavior that contributes to the maintenance of a low body weight 1. Some studies have reported that the age-adjusted and gender-adjusted incidence of AN is approximately 4.7 per 100,000 persons (95% Cl: 3.6-5.8) in the UK 3 and 8.3 per 100,000 persons (95% Cl: 7.1-9.4) in the USA 4. The age-adjusted incidence rate of AN showed a significant increasing trend only in females in late puberty-early adolescence 4,5. The mortality rate of AN is 5.1 deaths (95% Cl: 3.99-6.14) per 1,000 person-years; one in five individuals with AN who died had committed suicide 6. Cognitive behavioral therapy (CBT) 7,8, “the third wave” CBT 9, and family therapy 10,11 are reported as treatment approaches for AN. However, no clear primacy of one approach has been identified for AN 12. Treatment for AN is often arduous because its pathogenesis is not entirely understood. One important problem of AN is partial cognitive deficit. We reported in a previous study that patients with AN have a low intelligence quotient (IQ) 13. However, some studies have reported that patients with AN have an average IQ 14-16. What accounts for the difference in the results between these studies? One of the apparent differences is the patient's body mass index (BMI). We have reported a low IQ among AN patients with very low BMI (average 12.8) compared to that of patients in other reports (average BMI; 15.4-18.3) 13. These findings indicate that lower BMI with AN may decrease the patient's IQ. Therefore, the question of whether decreasing IQ in patients with AN with low BMI is the cause or effect is thought to be very important.

The National Adult Reading Test (NART) is a word-reading test (50 short words of irregular pronunciation) widely used in research and clinical practice as an estimate of premorbid intellectual ability 17. It has high construct validity as a measure of general intelligence and high levels of interrater and test-retest reliability. There are strong correlations between the NART and the Wechsler Adult Intelligence Scales (WAIS) performance IQ (PIQ) (*r* = .724) 18. Some studies have reported that patients with AN have a normal IQ as measured by the NART 19. However, there are no reports that compare the NART and the WAIS in the same patients with AN. The purposes of this study were as follows: to compare premorbid IQ with present IQ in patients with more severe AN-R and to investigate the relationship between decreasing IQ and symptoms in patients with severe AN-R.

## Materials and methods

### Participants

The patients were consulted for medical treatment at Kagoshima University Hospital. Volunteers were recruited by a notice board that was placed on Kagoshima University. All participants met with researchers, the study plan was explained in detail, and all participants signed informed consent forms. The inclusion criteria for patients were as follows: (1) patients met the Diagnostic and Statistical Manual of Mental Disorders Fourth Edition TR criteria for AN-R, (2) their body mass index (weight in kg/height in m^2^; BMI) was 15 or less, and (3) an examination was feasible. The exclusion criteria for patients were as follows: (1) had comorbidities, and (2) were less than 18 years old.

### Outcome measurement

#### Cognitive function assessments

The Wechsler Adult Intelligence Scale-Third Edition (WAIS-III) and the Japanese Adult Reading Test (JART) were used.

The WAIS-III is a neuropsychological assessment of intelligent quotient that provides four indices, each based on a different set of subtests (full-scale IQ (FIQ), verbal IQ (VIQ), performance IQ (PIQ), verbal comprehension (VC), perceptual organization (PO), working memory (WM), and processing speed (PS)), making it a more comprehensive conception of cognitive function 20. In this study, FIQ, VIQ, PIQ, VC, PO, WM, and PS were used.

The JART is the Japanese version of the National Adult Reading Test (NART) 21. The NART provides estimates of premorbid intelligence through the use of reading tasks with 50 English words. On the JART, the task was changed to Japanese Kanji words. This assessment can speculate on the premorbid IQ of dementia patients by the number of correct answers for 50 Japanese kanji idioms (e.g., “不如帰”, “自惚”).

### Psychological tests

The Eating Disorders Inventory-2 (EDI-2), Beck Depression Scale-2 (BDI-2) and State-Trait Anxiety Index (STAI) were used.

The EDI-2 is a 91-item self-report inventory instrument for assessing symptoms and psychosocial factors associated with eating disorders and includes 11 subscales (drive for thinness, bulimia, body dissatisfaction, ineffectiveness, perfectionism, interpersonal distrust, interceptive awareness, maturity fears, asceticism, impulse regulation, social insecurity). Items are rated on a 6-point scale, with responses ranging from never to always. In accordance with EDI-2 scoring procedures, item scores were recorded on a 4-point scale (0 to 3) to form subscale scores 22.

The BDI-2 is a 21-item self-report inventory that measures depressive symptoms such as sadness, pessimism, suicidal thoughts or wishes, tiredness or fatigue, loss of energy, and loss of pleasure, among others 23,24. Each item is scored on a scale from 0 to 3, with a higher score indicating more serious depressive symptoms and total scores ranging from 0 to 63.

The STAI is a 40-item self-report instrument that is widely used and extensively researched in the assessment of state and trait anxiety 25,26. State anxiety is regarded as having a transitory nature and is characterized by subjective feelings such as tension, apprehension and nervousness. Trait anxiety refers to relatively stable individual differences in anxiety proneness. Each item is rated on a 4-point Likert scale ranging from 1 to 4, with a higher score indicating more serious anxiety symptoms and total scores ranging from 20 to 80.

### Statistical analysis

SPSS Statistics 22 was used for statistical analysis. Comparisons of averages between groups (AN-R and control) were based on Student's t-test and two-way analysis of variance (ANOVA). Associations between IQ changes due to weight loss and psychological tests (EDI-2, BDI-2 and STAI) were estimated with correlation coefficients in the AN-R group.

### Ethical considerations

This study was approved by the Clinical Research Ethics Committee of Kagoshima University (registered No. 25-018). Written informed consent was obtained from all participants before participation.

## Results

Twenty-two participants were recruited (12 were AN-R patients; 10 were healthy controls). The participants were women, had a history of disease for more than one year, and had received different individualized treatment protocols. Table 1 shows the characteristics of the participants. The average BMI in AN-R patients and healthy controls was 12.65 and 19.82, respectively. There were significant differences in BMI and scores on the WAIS (FIQ, VIQ, PIQ and PO), JART (premorbid FIQ, premorbid VIQ and premorbid PIQ), EDI-2, BDI-2 and STAI (Table 1). In two-way ANOVA, there were significant interactions for the FIQ (F (1, 40) = 4.79, p < .05) and PIQ (F (1, 40) = 18.04, p < .01). Only in the AN-R group, a significant single main effect of time was evidenced for the FIQ (F (1, 40) = 16.40, p < .01) and PIQ (F (1, 40) = 34.15, p < .01) (Table 2). In the AN-R group, a significantly high positive correlation was found between changes in the PIQ and the body dissatisfaction subscale of the EDI-2 (Table 3).

## Discussion

This is the first study to compare premorbid IQ with present IQ in patients with more severe AN-R and conduct multiple investigations of the relationship between decreasing IQ and symptoms in severe AN-R using psychological tests. Previous studies in AN patients (average BMI 15.4-18.3) reported normal IQ using the WAIS-R or WAIS-III 14-16. However, in the present study, the full IQ and cognitive functioning (particularly PO) scores in patients with AN-R indicated almost borderline IQ. The difference between the results of the previous study and those of the present study was thought to be due to the difference in the BMI scores of the study participants. Furthermore, a previous study that administered the WAIS-III among patients with severe AN-R reported results that were similar to those of this study 13. In addition, previous studies have reported that IQ scores (FIQ, PIQ, and PO) recover significantly with weight re-gain 13. It is speculated that weight re-gain through CBT and nutritional treatment will restore the reduced IQ in patients with severe AN.

In this study, the JART showed significant differences when the premorbid FIQ, premorbid VIQ and premorbid PIQ in AN-R patients were compared with those in healthy controls. However, considering that the IQ score is defined as mean = 100 and SD = 15, the results of premorbid IQ in AN-R were normal 20. Therefore, these findings indicate that premorbid IQ might not be a risk factor for severe AN-R, although the number of patients in this study was limited. Future studies that include larger samples could address this limitation.

AN-R patients are dissatisfied with their figures and lose body weight 27,28. They decrease their food intake and over-exercise, even though they are facing a life crisis. In this study, there were no correlations between a drive for thinness and decreased IQ. This result was similar to that of a previous study 13. However, in the AN-R group, a strong positive correlation was found between the decreased PIQ and the body dissatisfaction subscale of the EDI-2 (Table 3). A person with a low PIQ has a slow information processing speed 20. It is difficult for the person to develop a greater understanding of larger concepts and grasp abstract content. In addition, people with low PIQ have low visual-spatial cognitive ability and difficulty doing multiple things at the same time. These findings raise the possibility that in patients with severe AN-R, an excessive decrease in body weight induces decreased PIQ; as a result, they have worse dissatisfaction with their body shape.

We have revealed that patients with severe AN-R have normal premorbid IQ and decreased postmorbid IQ; furthermore, their postmorbid PIQ is remarkably low. Therefore, given this decreased IQ, including PIQ, we should treat severe AN-R patients.

## Figures and Tables

**Table 1:** Clinical characteristics of the participants

	Control		AN-R		*t*	*p*	Cl
	Mean	SD	Mean	SD			
Age	32.40	9.65	32.17	14.45	-0.05	0.964	-11.05, 10.58
Education years	14.00	1.41	12.75	2.22	-1.60	0.126	-2.89, .39
BMI (kg/m^2^)	19.82	1.81	12.65	1.47	-10.26^*^	0.000	-8.62, -5.71
**WAIS-III**							
FIQ	103.20	10.26	81.00	13.37	-4.30^*^	0.000	-32.98, -5.71
VIQ	101.40	12.74	85.42	14.13	-2.76^**^	0.012	-28.06, -3.91
PIQ	103.50	8.55	78.92	10.72	-5.86^*^	0.000	-33.34, -15.83
VC	97.70	10.76	89.67	13.18	-1.54	0.138	-18.89, 2.82
PO	100.00	6.52	76.08	10.36	-6.32^*^	0.000	-31.81, -16.02
WM	100.00	14.16	87.58	14.51	-2.02^†^	0.057	-25.24, .40
PS	106.10	16.11	92.17	22.17	-1.65	0.114	-31.51, 3.64
**JART**							
premorbid FIQ	106.30	3.95	96.58	6.75	-4.20^*^	0.001	-14.57, -4.86
premorbid VIQ	108.60	4.65	96.67	7.55	-4.35^*^	0.000	-17.66, -6.21
premorbid PIQ	102.10	2.96	96.83	4.82	-3.01^*^	0.007	-8.92, -1.61
EDI-2	31.20	26.57	81.42	39.20	3.44^*^	0.003	19.76, 80.67
BDI-2	6.00	4.14	20.92	12.07	4.01^*^	0.001	6.93, 22.90
STAI-State	42.00	8.56	53.17	12.86	2.34^**^	0.030	1.23, 21.11
STAI-Trait	38.30	5.23	52.67	10.82	4.06^*^	0.001	6.89, 21.84

Control = 10, AN-R = 12; AN-R: anorexia nervosa restricting type; BMI: body mass index; WAIS-III: wechsler adult intelligence scale third edition; FIQ: full-scale intelligence quotient; VIQ: verbal intelligence quotient; PIQ: performance intelligence quotient; VC: verbal comprehension; PO: perceptual organization; WM: working memory; PS: processing speed; JART: japanese adult reading test; EDI-2: eating disorders inventory-2; BDI-2: beck depression inventory-2; STAI: state trait anxiety inventory.Results are expressed as mean ± SD; Cl, 95% confidence interval. ^†^*p* < .10, *p < .05, **p < .01.

**Table 2:** Two-way ANOVA (group×time) results

		Time		F-number		
		Pre	Post	Group	Time	Interaction
FIQ	Control	106.30 ± 3.95	103.20 ± 10.26	31.26^**^	10.71^**^	4.79^*^
	AN-R	96.58 ± 6.75	81.00 ± 13.37			
VIQ	Control	108.60 ± 4.65	101.40 ± 12.74	18.99^**^	8.29^**^	0.40
	AN-R	96.67 ± 7.55	85.42 ± 14.13			
PIQ	Control	102.10 ± 2.96	103.50 ± 8.55	43.09^**^	13.19^**^	18.04^**^
	AN-R	96.83 ± 4.82	78.92 ± 10.72			

^†^*p* < .10, *p < .05, **p < .01. AN-R: anorexia nervosa restricting type; FIQ: full-scale intelligence quotient; VIQ: verbal intelligence quotient; PIQ: performance intelligence quotient.

**Table 3:** The correlation between IQ change and psychological scale with weight loss

	FIQ	VIQ	PIQ
Age	.35	.41	.23
BMI	.13	.06	.26
EDI-2	.26	.24	.26
drive for thinness	.22	.17	.23
bulimia	.21	.21	.16
body dissatisfaction	.49	.42	.60^*^
ineffectiveness	.21	.25	.09
perfectionism	.45	.44	.46
interpersonal distrust	.17	.16	.16
interceptive awareness	-.06	-.09	-.06
maturity fears	.08	.07	.10
asceticism	.17	.16	.20
impulse regulation	.14	.14	.13
social insecurity	.01	.03	-.02
BDI-2	.01	.02	-.04
STAI-State	.20	.18	.19
STAI-Trait	.17	.17	.14

*p < .05. FIQ: full-scale intelligence quotient; VIQ: verbal intelligence quotient; PIQ: performance intelligence quotient; BMI: body mass index; EDI-2: eating disorders inventory-2; BDI-2: beck depression inventory-2; STAI: state trait anxiety inventory.
